# Monitoring Concrete Deterioration Due to Reinforcement Corrosion by Integrating Acoustic Emission and FBG Strain Measurements

**DOI:** 10.3390/s17030657

**Published:** 2017-03-22

**Authors:** Weijie Li, Changhang Xu, Siu Chun Michael Ho, Bo Wang, Gangbing Song

**Affiliations:** 1 Department of Mechanical Engineering, University of Houston, Houston, TX 77204-4006, USA; wli27@uh.edu (W.L.); siuchun.ho@gmail.com (S.C.M.H.); gsong@uh.edu (G.S.); 2 College of Mechanical and Electronic Engineering, China University of Petroleum, Qingdao 266580, China; chxu@upc.edu.cn; 3 Key Laboratory of Transportation Tunnel Engineering, Ministry of Education, Southwest Jiaotong University, Chengdu 610031, China

**Keywords:** reinforcement corrosion, reinforced concrete, acoustic emission, fiber Bragg grating

## Abstract

Corrosion of concrete reinforcement members has been recognized as a predominant structural deterioration mechanism for steel reinforced concrete structures. Many corrosion detection techniques have been developed for reinforced concrete structures, but a dependable one is more than desired. Acoustic emission technique and fiber optic sensing have emerged as new tools in the field of structural health monitoring. In this paper, we present the results of an experimental investigation on corrosion monitoring of a steel reinforced mortar block through combined acoustic emission and fiber Bragg grating strain measurement. Constant current was applied to the mortar block in order to induce accelerated corrosion. The monitoring process has two aspects: corrosion initiation and crack propagation. Propagation of cracks can be captured through corresponding acoustic emission whereas the mortar expansion due to the generation of corrosion products will be monitored by fiber Bragg grating strain sensors. The results demonstrate that the acoustic emission sources comes from three different types, namely, evolution of hydrogen bubbles, generation of corrosion products and crack propagation. Their corresponding properties are also discussed. The results also show a good correlation between acoustic emission activity and expansive strain measured on the specimen surface.

## 1. Introduction

Reinforced concrete (RC) structures constitute the majority of civil infrastructures of the world. Corrosion of concrete reinforcement members has been recognized as one of the predominant deterioration mechanisms for steel reinforced concrete structures. The two major factors responsible for corrosion of steel reinforcement are carbonation of concrete and chloride penetration. Of the two, chloride induced corrosion is a far more serious problem. Under chloride attack, the passivation of steel within the concrete is broken down and indicates the initiation of reinforcement corrosion. The corrosion products are usually two to six times more voluminous than the original steel consumed [1], resulting in the generation of expansive pressure exerted by corrosion products to the surrounding concrete. Damage due to reinforcement corrosion occurs in the form of cover cracking, reduction of the steel cross-sectional area, and deterioration of a concrete–steel interface bond [2]. Failure of the structures leads to heavy economic loss, environmental pollution and increased risks of injury. It is therefore of paramount importance to monitor and characterize steel corrosion in RC structures and assess their performance and safety.

Structural health monitoring for concrete structures has attracted a lot of attention [3,4,5]. A wide variety of techniques have been reported in the literature that may be suitably employed for monitoring and locating corrosion nondestructively. Some commonly used reinforcement corrosion monitoring techniques are electrochemical measurements [6,7,8], ultrasonic testing methods [9,10], radiography [11] and infrared thermography [12,13]. Methods based on electrochemical measurements are the most popular ones, which directly take advantage of the electrochemical nature of steel corrosion reaction. They can only provide information about the possibility of a corrosion event, and fail to describe the rate of corrosion and the accumulated amount of corrosion. Ultrasonic testing relies on the variation of wave propagation properties due to structural damage caused by steel corrosion, and is limited by its short propagation distance. Radiography relies on the passage of radiation (i.e., X-rays or gamma rays) through the structure and the consequent degrees of attenuation depending on the state of the structure (e.g., defects). The accuracy of radiography techniques is high; however, to accomplish radiographic measurements, both sides of the structure in question must be accessible. Depending on the type of radiation used, additional safety precautions are required for the operator. Infrared thermography may be a good choice for corrosion monitoring due to the strong correlation between the apparent diffusion coefficient of chloride ion and the heat dissipation characteristics of concrete. However, the interpretation of infrared thermography is only qualitative, incapable of quantitatively indicating the chloride content inside the concrete.

Concrete expansion and cracking are among the most significant and obvious physical features related to concrete deterioration due to reinforcement corrosion, and thus monitoring of these two features is critical for evaluating the safe performance of reinforced concrete structures. These two features can be readily detected using acoustic emission (AE) techniques and fiber optic sensing, both of which have recently emerged as new methods to measure and characterize corrosion in concrete structures.

Acoustic emission is comprised of high frequency elastic waves or stress waves generated when there is a rapid release of energy within a material that is undergoing deformation [14,15,16]. In concrete structures, as corrosion products are formed around steel reinforcement, pressure is built up in the surrounding concrete. When the pressure is high enough to break the interface bonding, micro-cracking and crack growth will induce stress waves that are immediately captured by AE sensors mounted on the concrete surface. Corrosion characteristics can be interpreted from AE parameter analysis. Li et al. showed that AE technique can be used in rebar corrosion detection [17]. Idrissi and Limam demonstrated a high correlation between the progressive characteristics of acoustic emission and the current density of corrosion [18]. Kawasaki et al. identified different corrosion stages such as onset of corrosion and nucleation of corrosion-induced cracking, all through AE parameter analysis [19]. The results were further verified by comparing with results from scanning electron micrograph and electron probe micro analyzer.

Fiber optic sensors carry numerous advantages over traditional sensors, such as small size, immunity to electromagnetic interference, corrosion resistance, and real-time monitoring [20,21]. Therefore, they are very suitable for corrosion monitoring in reinforced concrete structures. Extensive investigation has been conducted concerning the suitability of fiber optic sensors to monitor strain in reinforced concrete structures under corrosive environment. Grattan et al. used a fiber Bragg grating (FBG) strain sensor to monitor the production of rusts during corrosion process [22]. Zheng et al. reported wrapping an FBG around a reinforcement member to capture surface strains of the member due to corrosion-induced expansion [23]. Zhao et al. examined the feasibility of a Brillouin optical time domain analysis (BOTDA) distributed sensing technique for measuring steel corrosion expansion [24]. Mao et al. investigated the combination of BOTDA and FBG sensors for monitoring corrosion expansion cracking [25]. Li et al. examined the rebar corrosion using the combined carbon fiber and FBG active thermal probe [15]. McCague et al. designed a novel corrosion sensor based on birefringent photonic crystal fibers for pressure/force measurement [26]. Leung et al. proposed a steel corrosion detection method using a simple light reflection technique, in which one end of the fiber was coated with an iron thin film to interrogate steel corrosion [27]. Li et al. devised a metal loss detector for corrosion measurement based on fiber optic macro-bend light loss [28].

In the present study, an approach based on the combination of the AE technique and FBG strain measurements is presented with the aim to monitor the progression of reinforcement expansion and concrete cracking due to reinforcement corrosion in concrete structures. Acoustic emission measurement helps to detect corrosion initiation and crack growth while FBG strain measures the extent of corrosion and identify time of cracking. Such a combined approach has not yet been reported in the literature, and the results of such a study can provide a correlation between acoustic emission events and concrete surface strains. A reinforced mortar specimen was prepared and an accelerated corrosion test was performed in laboratory to verify the proposed method. The rest of the paper is organized as follows: Section 2 briefly describes the corrosion-induced cracking mechanism and Section 3 illustrates the experimental setup of the corrosion tests. Experiment results are shown and discussed in Section 4 and conclusions are drawn in Section 5.

## 2. Corrosion-Induced Cracking Process

Corrosion of steel reinforcement members leads to the desolvation of metallic iron into corrosion products as a result of the oxidation process. This process gives rise to an increase in volume, which, depending on the level of oxidation, may be up to about six times the original iron volume. The composition of the expansive corrosion products usually exist in the form of $\;\left\{a\cdot \mathrm{Fe}{\left(\mathrm{OH}\right)}_{2}+b\cdot \mathrm{Fe}{\left(\mathrm{OH}\right)}_{3}+c\cdot H_{2}O\right\}$, where *a*, *b* and *c* are the variables that depend on the alkalinity of the pore water solution of concrete, the oxygen supply and the moisture content.

**Figure 1:**
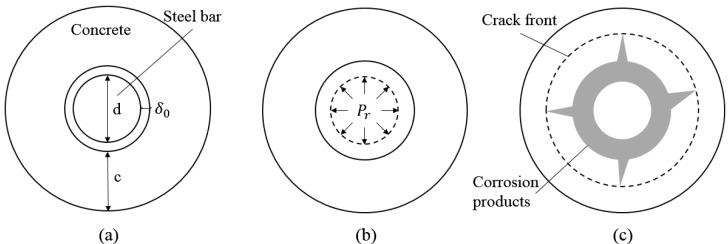
Schematic diagram of corrosion-induced concrete cracking process: (**a**) initial unrestrained reinforced concrete; (**b**) internal expansive pressure for concrete in restrained condition; (**c**) corrosion products deposited within open cracks.

According to the corrosion cracking model for reinforced concrete described by Zhao and Jin [29], the steel corrosion process can be divided into three stages, namely, the free expansion of the corrosion products, stress build-up in the concrete cover and the cracking of concrete cover. Figure 1a illustrates the initial unrestrained condition for a reinforced concrete cylinder with a steel bar of initial diameter d and a clear cover of thickness  c. There exists a porous zone around the steel–concrete interface that has a mean thickness of $\;\delta_{0}$. Once the steel starts corroding, the corrosion products will expand freely until the porous zone is filled and then the uniform expansive pressure $P_{r}$ begins to develop on the surrounding concrete wall. As the corrosion progresses further, the expansive pressure will induce tensile stresses and strains on the internal concrete wall, as shown in Figure 1b. In the meantime, the corrosion products will also be restrained and condensed by the surrounding concrete. When the hoop tensile stress at any part of an inner circumference exceeds the tensile strength of the concrete, the corrosion-induced cracks will occur firstly at the steel–concrete interface and then gradually expand towards the outer cover surface, as depicted by Figure 1c. The concrete cracking process due to reinforcement corrosion will be monitored by an acoustic emission technique, whereas the concrete expansion process will be measured by FBG strain sensor.

## 3. Experimental Setup

To verify the proposed approach for reinforcement corrosion monitoring through a combined AE and FBG detection system, a cylindrical reinforced mortar specimen was prepared and an accelerated corrosion test was conducted under laboratory conditions. Both AE transducers and the FBG sensor were used in the same corrosion specimen so as to investigate the correlation between acoustic emission events and concrete surface strain.

### 3.1. Preparation of Specimen

**Figure 2:**
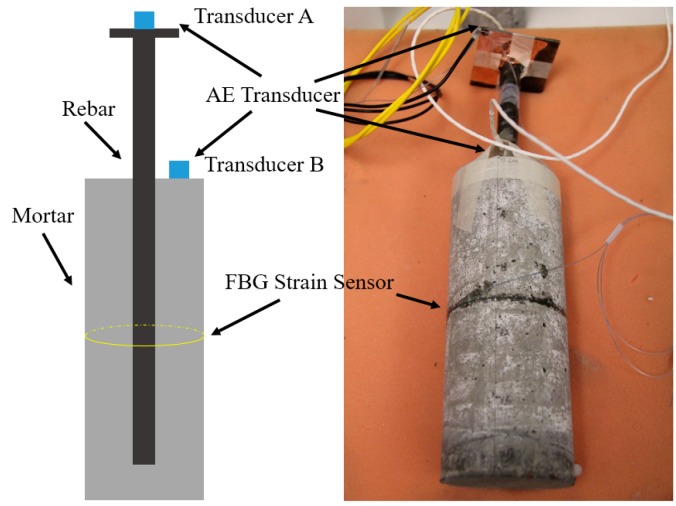
Reinforced mortar specimen and sensor installation.

The cylindrical reinforced mortar specimen was 80 mm in diameter and 200 mm in height, as shown in Figure 2. The cement used was ordinary Portland cement (OPC) and the cement, water, sand mix ratio was 1:0.5:2 by weight. One deformed rebar 16 mm in nominal diameter was embedded longitudinally into the center of the mortar cylinder. The rebar was 300 mm long with one end embedded approximately 20 mm away to the bottom of the mortar cylinder. A small steel plate was welded to the exposed end of rebar for the convenience of AE transducer installation. The mortar was removed from the PVC mold one day after being casted. It was cured in moist environment for seven days and dried in air for another 20 days under room temperature.

### 3.2. Setup of Monitoring Systems

Two AE transducers were mounted on the specimen. One transducer was on the top of the steel plate, and the other was on the top surface of the mortar, as shown in Figure 2. The purpose of such an arrangement was to study the characteristic of AE signal propagating via different media. Lubricant grease was applied on the mounting surface before attaching AE transducers in order to ensure good contact for signal detection between transducer surface and specimen surface. The AE transducers were then mounted on the surface and tightened using adhesive tape. An FBG strain sensor (central wavelength: 1544.878 nm, strain coefficient: 1.15  pm/με) was circumferentially bonded around the mortar surface in the middle cross section, as depicted in Figure 2. The middle section was chosen in order to record a more uniform expansion. The FBG strain sensor was pre-stressed about 300 pm. A thin layer of epoxy (Epoxy GEL, Devcon, Danvers, MA, USA) of 17 MPa strength was then applied for sensor protection. To eliminate the influence of temperature variation, an FBG temperature sensor (central wavelength: 1549.335 nm, temperature coefficient: 10.3 pm/°C was also connected in line with the strain sensor. The FBG temperature sensor was prepared by putting a bare FBG into a small stainless steel tube, followed by sealing both ends of the tube using epoxy, to ensure that the FBG is only sensitive to temperature variation.

**Figure 3:**
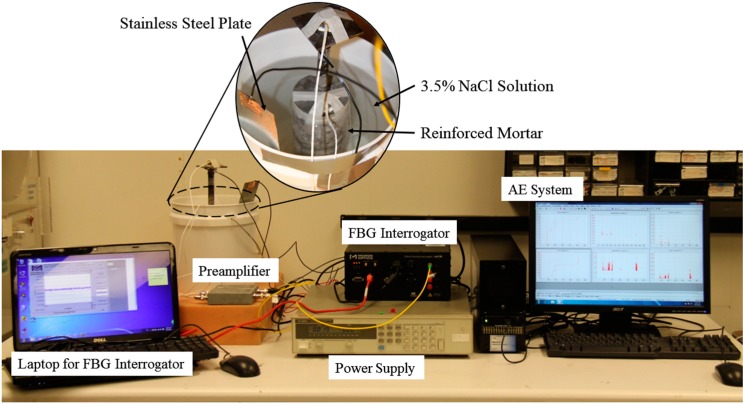
Reinforced mortar specimen and sensor installation.

The corrosion monitoring systems consists of two separate systems, namely, the AE corrosion monitoring system and the FBG strain monitoring system. The layout of these two systems are depicted in Figure 3. The AE instrumentation made up of AE transducers, preamplifiers and an AE system (Micro-ΙΙ Digital AE System, Physical Acoustic Corp. (PAC), Princeton Junction, NJ, USA). The transducers used were R15 type, which resonate at 150 kHz. The sampling frequency for the recording waveforms was 5 MHz. The threshold level was set at 40 dB, which is sensitive enough to capture corrosion induced signal while allowing the acquisition of all the significant data. The waveforms was amplified with 40 dB gain by a preamplifier. The AE waveforms and parameters, such as events, hits, counts, energy and duration, were recorded on the computer, and can be exported as ASCII files for further processing.

For the FBG strain monitoring system, the wavelength variation due to corrosion expansion was interrogated by an FBG interrogator (sm130, Micron Optics Inc., Atlanta, GA, USA). The interrogator was connected to a laptop via Ethernet, and the wavelength data was stored in the laptop with a saving frequency of one sample every 100 s.

### 3.3. Pencil Lead Break Test

The attenuation rate of acoustic emission signal transmitted in concrete and steel materials are different. To check the sensitivity of each sensor, a pencil lead break test was conducted on the mortar specimen. The test consists of breaking a 0.5 mm diameter pencil lead by pressing it against the specimen surface. The testing points were along the longitudinal direction and the spacing between these points was 40 mm. Thus, there will be six testing points in total.

### 3.4. Accelerated Corrosion Test

To corrode the reinforced mortar in a reasonable amount of time, an accelerated corrosion test was performed. The specimen was placed in a bucket filled with 3.5% NaCl solution, which was used to simulate seawater. The specimen was immersed in the NaCl solution for three days before a constant current was applied. The constant current was set at 80 mA by a power supply. The anode was connected to the rebar and the cathode was connected to the sacrificial stainless steel plate, as shown in Figure 3. The corrosion test was conducted in a quiet room. In addition, the bucket was placed on a sponge cushion to reduce the noise coming from the environment.

## 4. Results and Discussion

### 4.1. Results from Pencil Lead Break Test

**Figure 4:**
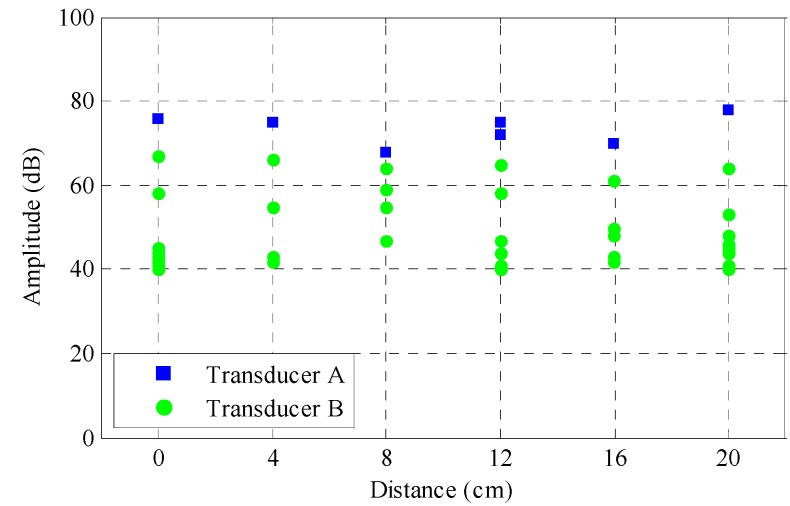
Amplitude from both transducers at different locations.

Attenuation of the generated acoustic emissions was considered as having a major influence on the sensitivity of the transducer and accuracy of the collected data. A pencil lead break test was used to produce similar AE events and check the sensitivity of each transducer. The signal amplitude received from both transducers during the pencil lead break test is shown in Figure 4. Each column in the graph represents a different pencil lead breaking location starting from the top to the bottom of the specimen with increments of 40 mm. As can be seen, both transducers are quite sensitive to the AE source located at any place of the specimen, capable of reaching 60 dB in amplitude. Transducer A, which was mounted on the reinforcement, is more sensitive than Transducer B, which was attached to mortar. The difference of highest amplitude between the transducers is approximately 10 dB.

**Figure 5:**
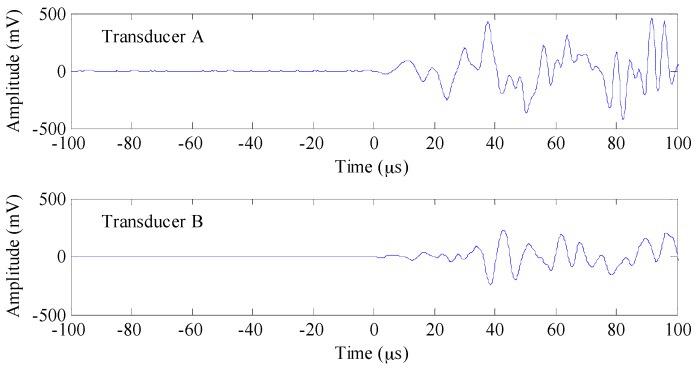
Waveforms from pencil lead break tests for both transducers.

Figure 5 shows the recorded waveforms corresponding to events of highest amplitude at the same testing location. The amplitude from Transducer A is much greater than that from Transducer B. The waveform that propagated through the mortar was more attenuated as compared to the one that propagated through the steel reinforcement, that is to say, the mortar provides greater damping effect on the acoustic signal than the steel reinforcement. The pencil lead break test confirms that attenuation of the AE signal transmitted in mortar is greater than that in reinforcement.

### 4.2. Acoustic Emission Study

**Figure 6:**
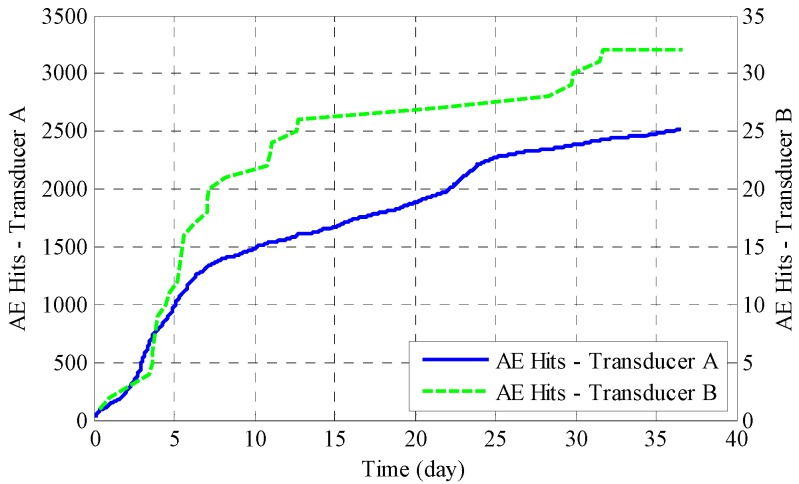
AE hits during the accelerated corrosion test.

AE hits from both transducers during accelerated corrosion of the reinforced mortar specimen are shown in Figure 6. The hits number measured by Transducer B is far less compared to Transducer A, indicating that the mortar attenuated the AE signal at a much greater rate than did the steel, much in the same way as in the earlier pencil-break tests. Since only an insignificant amount of AE events was captured by Transducer B (only 30 events), the interpretation from Transducer B will be very inaccurate. Thus, in the following analysis, we will focus our discussion on results obtained from Transducer A. The curves from Transducer A show a high similarity with the phenomenological model of reinforcement corrosion in marine environments [30]. The corrosion activity is strong in the early stage, followed by an evident decrease in the corrosion rate after around seven days. In this period, the flow of oxygen and water, which are essential to steel corrosion, is inhibited by the buildup of corrosion products, thus slowing down the corrosion process. The corrosion proceeded further after 22 days with the assistance of anaerobic corrosion until cracks were observed on the concrete surface on around the 36th day.

**Figure 7:**
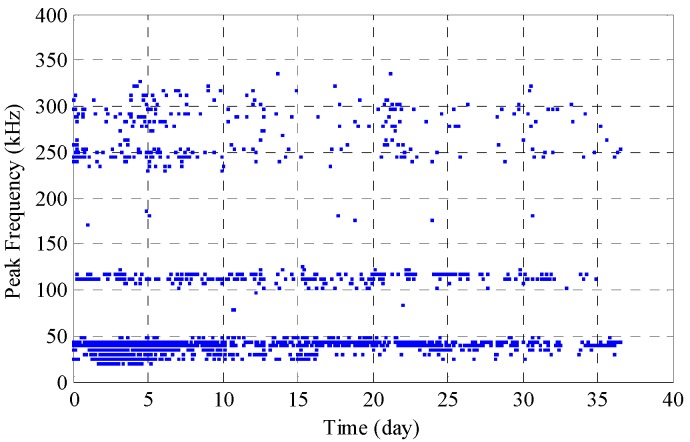
Peak frequency content from Transducer A.

**Figure 8:**
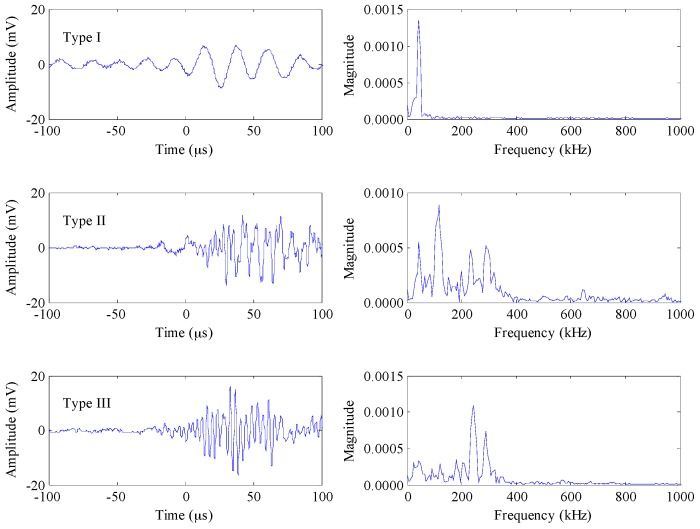
Typical waveforms of different types and their fast Fourier transform (FFT).

Peak frequency is the frequency with the maximum magnitude as determined by a fast Fourier transform (FFT) of the time domain signal. The peak frequency can serve as a good indicator for different AE sources arising from reinforcement corrosion. Other indicators of frequency, like average or central frequency, share the same properties. Figure 7 shows the peak frequency corresponding to each hits from Transducer A. It can be seen that the population can be divided into three major parts: one below 50 kHz (denoted as Type I), one around 110 kHz (denoted as Type II) and another part with peak frequency higher than 240 kHz (denoted as Type III). Specifically, the majority (75%) of the hits exhibit peak frequency lower than 50 kHz, while 11% are around 100 kHz and 14% are scattered in the highest band above 200 kHz. These three types of frequency bands are in close relation to different AE sources due to reinforcement corrosion. Mazille and Rothea [31] stated that bubble evolution in the liquid medium gave low frequency AE signals (~50 kHz); therefore, Type I can be attributed to the evolution of hydrogen bubbles from electrochemical reaction between anode and cathode. As suggested by Yoon’s work [32], AE signals from micro-cracking and macro-cracking are of short duration and of relatively high frequency (180 kHz to 350 kHz), and the AE signals associated with debonding are of longer duration and their frequencies peak at 120 kHz. Li et al. [17] concluded that the peak frequencies for a typical concrete crack signal were high frequencies, ranging from 275 kHz to 350 kHz. It is reasonable to state that Type II appears to correspond to the generation and movement of corrosion products and reinforcement/mortar interface debonding while Type III peak frequencies comes from micro-crack development and crack propagation. All of these three types of peak frequencies occurred for the whole span of the corrosion test. Therefore, in our case, we can surmise that the process of final mortar cover cracking is an accumulative process, which is comprised of a large amount of micro-cracking events before final cover cracking occurs. Figure 8 presents typical waveforms of these three types and their corresponding fast Fourier transform (FFT).

**Figure 9:**
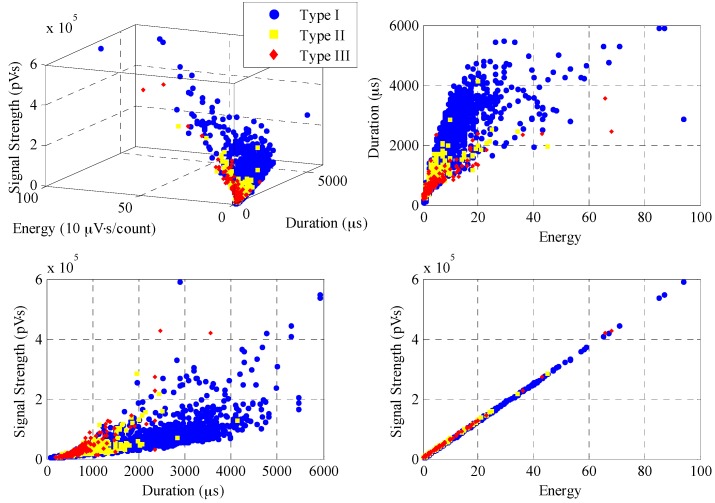
3D cross-plot of duration, energy and signal strength and its perspective plots.

The cross-plot is an effective technique to analyze the features of an entire AE signal for the purpose of distinguishing signal characteristics. Figure 9 shows the results of a 3D cross-plot of duration, energy and signal strength of AE signals obtained from Transducer A. The circles represent AE events from Type I and squares indicate AE events from Type II, whereas diamonds are those from Type III. Duration is defined as the time from the first threshold crossing to the end of the last threshold crossing of the AE signal. Energy is defined as the integral of the rectified voltage signal over the duration of the AE hit. Signal strength is defined as the measured area under the amplitude time envelope. It can be noted that Type I AE events are spread over the plot, characterized by long duration, high energy and strong signal strength and Type II AE events feature shorter duration, lower energy and weaker signal strength, whereas Type III AE events are more tightly clustered and have the smallest value of the three parameters above. Overall, these three types of AE events, due to reinforcement corrosion, can be easily recognized.

### 4.3. FBG Strain Measurement

**Figure 10:**
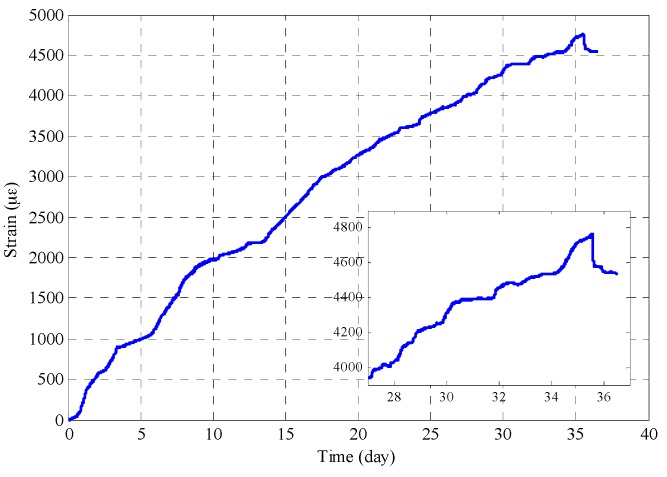
Strain history from FBG strain measurement.

**Figure 11:**
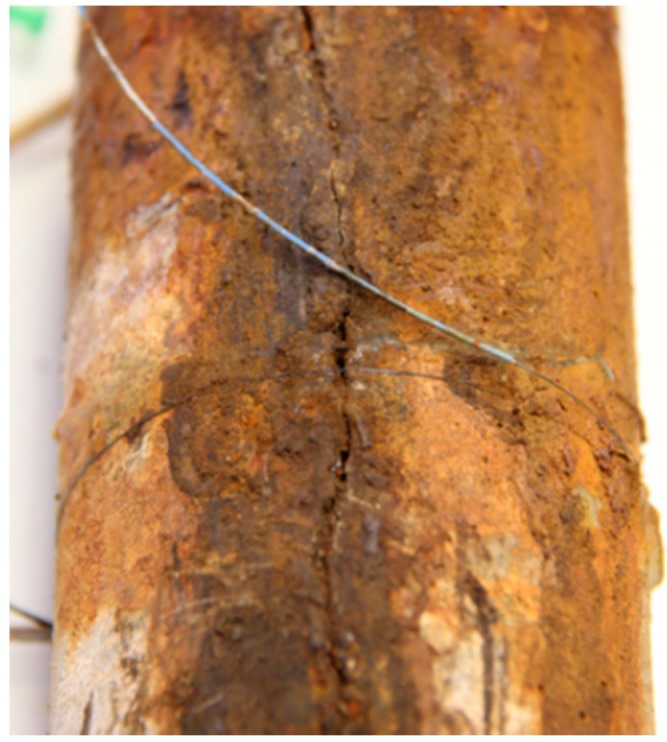
Illustration of cracking of the specimen.

The circumferential strain variation of the reinforced mortar was measured using an FBG strain sensor. Measurement commenced the moment that the current was applied all the way to when the specimen cracked. The strain data over the time span of about 36 days is shown in Figure 10. The original wavelength variation was converted to strain variation by adopting a strain sensitivity coefficient of 1.15  pm/με, which was calibrated beforehand. As can be seen, the strain increased with time as the corrosion products accumulated and exerted internal pressure on the mortar. The strain grew approximately 1000 με every five days, and slowed down to about 500 με every five days in the later period. The change in the strain rate can be attributed to the formation of cracks, which provides new space for corrosion products. The corrosion products fills the crack before building up stress on the mortar when cracks were generated. Notice that, after 24 days, the strain curve exhibited a jagged variation that results from the redistribution of the corrosion products into the widening cracks as the crack front propagates further into the cover. This period can be identified as the crack propagation stage. The embedded plot partially shows an enlarged view of the jagged curve. The crack reached the outer surface on around the 36th day and was marked by a sudden drop in strain reading. After many days of internal pressure buildup from the accumulation of corrosion products, the whole mortar cylinder has become stressed. When the pressure in the mortar surface exceeds its tensile strength, surface cracking occurs and the pressure on the mortar surface suddenly releases, resulting in a drop in stress or strain on the surface. It is noteworthy that surface microcracking occurred throughout all of the stages as long as the stress exceeds the tensile strength of the concrete. As reinforcement corrosion continued, microcracks developed into macrocracks. The macrocrack, which had a width of 1.391 mm, is shown in Figure 11. The crack width estimated from strain measurement was 1.141 mm (4540 με×πD), which shows good agreement with results from direct measurement.

The above results showed that FBG strain measurement is effective in monitoring reinforcement corrosion induced mortar expansion and cracking. For the case investigated in this study, a single strain curve provides corrosion information from these three aspects. First, the tensile strain around the mortar specimen due to expansive pressure exerted by the formation of corrosion products was continuously monitored. Second, the crack initiation and propagation period, featured by notable jagged variation of strain curve, can be easily recognized. Third, the moment of the first appearance of macrocracks on the mortar surface was detected and the crack width can be estimated from strain value. In future work, more samples will be tested and additional experiments will be designed to validate the efficacy of FBG strain measurement.

### 4.4. Discussion

**Figure 12:**
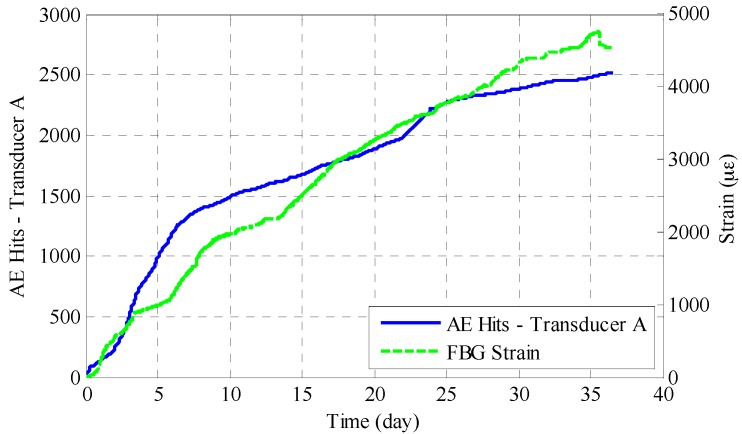
Simultaneous evaluation of acoustic emission activity and strain measurement.

Figure 12 is a plot that shows both acoustic emission hits and FBG strain measurement. These two different corrosion monitoring methods interpret the phenomenon of reinforcement corrosion from two different aspects; one is from acoustic emission aspects and the other is from expansive strain development aspects. As can be seen, the AE hits and FBG strain show a similar trend and confirm that the level of acoustic emission activity and the development of surface strain generally had a positive correlation as the corrosion of the reinforced concrete progressed. This agreement is qualitative, and, for a more detailed understanding and analysis of the AE signals, the adoption of advanced signal processing techniques and pattern recognition algorithms would be helpful.

The presented integrated corrosion monitoring technique was performed under controlled laboratory conditions. As for field application of the integrated corrosion monitoring technique, there will be many practical challenges. Since the reinforced concrete structure is generally large in size, surface mounting AE sensors may reduce the sensitivity and efficacy of the AE sensors. Alternatively, we recently proposed the low-cost, embedded smart aggregate as a type of embedded AE sensor [16]. The embedded AE sensors can be strategically installed at the locations that are prone to damage in order to increase the efficacy of the AE method. As for the FBG strain measurement, the authors will instrument the FBG sensors on the reinforced concrete samples in such a way that a ring shaped cavity is created around the rebar and FBG sensors are installed within the cavity. In this way, the stress transfer in circumferential direction is ensured and the fragile FBG sensors are well-protected from external interruption during construction.

## 5. Conclusions

In this paper, we present an integrated monitoring technique for reinforced concrete corrosion based on the novel coupling of acoustic emission and FBG strain sensing. A reinforced mortar was prepared and the corrosion process was accelerated through the injection of constant current. The setup allowed the study of corrosion characteristics at different stages. Corrosion monitoring can be divided into two aspects: the corrosion initiation and cracking propagation can be captured by an acoustic emission system, whereas the mortar expansion due to corrosion product generation will be monitored by a fiber Bragg grating strain sensor.

It was found from the pencil lead break test that the attenuation of AE signal transmitted in mortar material is greater than that in steel material. Analysis from AE hits show that the hit history resembles the phenomenological model of reinforcement corrosion loss in a marine environment. AE signals are generated from three types of sources, including hydrogen bubble evolution, generation and movement of corrosion products, and micro-crack development and crack propagation. These three types of AE sources distinguish themselves in parameters like duration, energy and signal strength. Through continuous monitoring of tensile strain using FBG strain sensors, crack initiation and the propagation period were identified, and the time of concrete cover cracking was detected. Good agreement was obtained between acoustic emission activity and expansive strain measured on the specimen surface. Specifically, a similar trend in AE activity and strain measurement was observed as corrosion progressed. The AE events characterize the corrosion development based on corrosion induced AE events, and FBG strain measurement characterized the corrosion in terms of concrete expansion induced by rebar corrosion. Results have shown that the integration of these two nondestructive techniques is promising for reinforced concrete corrosion monitoring and characterization.
